# Effects of Cancer, Chemotherapy, and Cytokines on Subjective and Objective Cognitive Functioning Among Patients with Breast Cancer

**DOI:** 10.3390/cancers13112576

**Published:** 2021-05-24

**Authors:** Vincent Chin-Hung Chen, Chin-Kuo Lin, Han-Pin Hsiao, Bor-Show Tzang, Yen-Hsuan Hsu, Shu-I Wu, Robert Stewart

**Affiliations:** 1 Department of Psychiatry, Chang Gung Medical Foundation, Chiayi Chang Gung Memorial Hospital, Chiayi 613016, Taiwan; cch1966@gmail.com (V.C.-H.C.); hanpin.hsiao@gmail.com (H.-P.H.); 2 School of Medicine, Chang Gung University, Taoyuan 33302, Taiwan; 3 Division of Pulmonary Infection and Critical Care, Department of Pulmonary and Critical Care Medicine Chang Gung Memorial Hospital, Chiayi 61306, Taiwan; lingh@cgmh.org.tw; 4 Graduate Institute of Clinical Medicine Sciences, College of Medicine, Chang Gung University, Taoyuan 33302, Taiwan; 5 Department of Biochemistry, School of Medicine, Chung Shan Medical University, Taichung 40201, Taiwan; bstzang@csmu.edu.tw; 6 Clinical Laboratory, Chung Shan Medical University Hospital, Taichung 40201, Taiwan; 7 Department of Psychology, National Chung Cheng University, Chiayi County 62102, Taiwan; oliviayhh@gmail.com; 8 Center for Innovative Research on Aging Society (CIRAS), National Chung Cheng University, Chiayi County 62102, Taiwan; 9 Department of Medicine, Mackay Medical College, New Taipei City 25245, Taiwan; 10 Department of Psychiatry, Mackay Memorial Hospital, New Taipei City 251020, Taiwan; 11 Department of Psychological Medicine, King’s College London, London SE5 8AF, UK; robert.stewart@kcl.ac.uk; 12 South London and Maudsley NHS Foundation Trust, London SE5 8AF, UK

**Keywords:** cancer, chemotherapy, subjective and objective cognitive functioning, neuropsychological testing, cytokines, inflammation

## Abstract

**Simple Summary:**

Although cognitive impairments have been complained about in patients with breast cancer who underwent chemotherapy, recent research has described possible neurocognitive decline prior to the start of chemotherapy and suggested that inflammatory cytokines may also have been involved. However, inconsistencies have been found in correlations of cognitive impairments with cancer, chemotherapy, and peridiagnostic cytokine levels. This cross-sectional study aimed to examine associations of cognitive functions and levels of cytokines in patients with newly- diagnosed breast cancer before chemotherapy, those that were 3 to 9 months after completing chemotherapy, and non-cancer controls, adjusting for baseline intelligence quotient, mood, and fatigue. We found that the performance in semantic association of verbal fluency in patients post chemotherapy might be affected by the status of cancer, IL-13, and anxiety. Our results indicated that verbal fluency and anxiety may be important when considering relevant psychosocial managements or prophylactic interventions for cognitive preservation associated with regulations in cytokines.

**Abstract:**

Background: We aimed to investigate the associations of breast cancer (BC) and cancer-related chemotherapies with cytokine levels, and cognitive function. Methods: We evaluated subjective and objective cognitive function in BC patients before chemotherapy and 3~9 months after the completion of chemotherapy. Healthy volunteers without cancer were also compared as control group. Interleukins (IL) 2, 4, 5, 6, 10, 12p70, 13, 17A, 1β, IFNγ, and TNFα were measured. Associations of cancer status, chemotherapy and cytokine levels with subjective and objective cognitive impairments were analyzed using a regression model, adjusting for covariates, including IQ and psychological distress. Results: After adjustment, poorer performance in semantic verbal fluency was found in the post-chemotherapy subgroup compared to controls (*p* = 0.011, *η*^2^ = 0.070); whereas pre-chemotherapy patients scored higher in subjective cognitive perception. Higher IL-13 was associated with lower semantic verbal fluency in the post-chemotherapy subgroup. Higher IL-10 was associated with better perceived cognitive abilities in the pre-chemotherapy and control groups; while IL-5 and IL-13 were associated with lower perceived cognitive abilities in pre-chemotherapy and control groups. Our findings from mediation analysis further suggest that verbal fluency might be affected by cancer status, although mediated by anxiety. Conclusions: Our findings suggest that verbal fluency might be affected by cancer status, although mediated by anxiety. Different cytokines and their interactions may have different roles of neuroinflammation or neuroprotection that need further research.

## 1. Introduction

Breast cancer (BC) is becoming the most prevalent cancer worldwide. The annual incidence of BC is over 45 per 100,000, and it is prevalent in more than 600,000 people per year [1]. With advances in treatments (e.g., chemotherapy, immunotherapies), BC survival rates have increased [2,3]; however, systemic chemotherapy can cause adverse reactions [3]. Although severe neurotoxic events are rare, risks of cognitive decline have varied in their estimates from 15~60% to the extent of interfering with the quality of everyday life [3,4,5,6,7]. Specifically, impairments in attention, concentration, learning, processing speed, and executive functioning have been reported [3]. Cognitive decline in BC may of course have multiple other underlying causes, including fatigue, sleep disturbance, psychological distress, or effects from other medication [8]. Although an up to 8-fold elevated risk of cognitive complaints was reported in BC patients who had undergone chemotherapy than those receiving other treatments [9], recent research has reported that 21–33% of BC patients experience neurocognitive decline prior to the start of chemotherapy [8]. Therefore, other factors might also be involved.

In this respect, there have been investigations of the relationship of cytotoxic agents, inflammatory cytokines, and cognitive impairments before [10], during [11,12,13], and after [14] BC chemotherapy [15]. Ganz et al. in a prospective study of BC patients after initial surgery, found a higher level of soluble tumor necrosis factor receptor type II (sTNFRII) among patients who received chemotherapy than in those who did not; this was correlated with initial subjective cognitive complaints. Declines in sTNFRII over years were associated with fewer complaints afterwards [14]. On the other hand, Patel et al. described reduced memory function in newly-diagnosed and pre-treatment BC compared to healthy controls, and this was associated with higher levels of pre-treatment sTNFRII [10]. Cheung et al. further reported a correlation between higher plasma IL-1β and poorer response speed; and higher IL-1β, IL-6, and more severe subjective cognitive complaints during chemotherapy receipt. Higher IL-4 was associated with better performances in response speed and fewer subjective complaints [11].

Inconsistencies have been found in correlations of cognitive impairments with cancer status, chemotherapy, and peridiagnostic cytokine levels. No previous studies have adjusted for baseline intellectual functions [16]. We thus designed this cross-sectional study aimed to examine associations of self-perceived and objective cognitive functions, cancer status, and levels of cytokines in BC patients before receiving chemotherapy and 3 to 9 months after completing chemotherapy, incorporating comparisons with non-cancer controls. Baseline intelligence quotient, mood, and fatigue were also evaluated.

## 2. Methods

### 2.1. Participants

BC adult patients diagnosed with Stage I to Stage III invasive breast cancer without metastasis from the oncology clinic at a medical center located in Southern Taiwan were invited to participate. Our non-cancer controls were demographically 1:1 matched female volunteers within 5 years of age of each index patient. Participants were excluded if they were currently pregnant, unable to read/write, had histories of developmental delay, severe visual impairments, or major neurological diseases (e.g., stroke, movement disorders, epilepsy, multiple sclerosis, head injury with loss of consciousness or neurological sequela, or other lesions from central nervous system). Subjective and objective neuropsychological assessments were administered to controls, and to newly diagnosed cases before the start of their chemotherapy (categorized as the pre-chemotherapy (pre-C/T) group). Patients from the post-chemotherapy (post-C/T) group were those who had completed chemotherapy for 3 to 9 months (average: 4.2 months). A total of 70 and 36 participants were categorized in the pre-C/T and post-C/T group, respectively. The Research Ethics Committee at Chiayi Chang Gung Memorial Hospital approved this study (IRB number: 201700255B0) on 1 June 2017. Written informed consent was obtained from all participants before entering the study.

### 2.2. Measures

Subjective and objective neuropsychological measures were administered by research assistants supervised by a psychiatrist and clinical psychologists on weekly basis. Details of measures used are described elsewhere [17]. In short, Taiwanese versions of the assessments comprised: (i) the Wechsler Adult Intelligence Scale, Third Edition (WAIS-III) [18] to estimate intelligence; (ii) the Block Design subtests to test visuospatial functions; (iii) the Digit Symbol Substitution to measure processing speed; (iv) the Digit Span [18] and the first part of the Taiwanese Color Trails Test (CTT1) [19] to assess attention; (v) the second part of the Color Trails Test (CTT2), the Semantic Association of Verbal Fluency Test (SFT) [20], and the Orthographical Fluency Test (OFT) [20], to examine executive functioning; vi) the Word List subtest of the Wechsler Memory Scale, Third Edition [21] to evaluate memory function (the two single-trial tasks were used to test prospective memory) [22]. Higher scores on all above tests indicated better cognitive performance, apart from the CTT1 and CTT2, where higher completion times indicated poorer performance.

We used the Functional Assessment of Cancer Therapy Cognitive Function Version 3 (FACT-Cog) [23] to evaluate self-perceived cognitive function. The FACT-Cog comprises four subscales: Perceived Cognitive Impairment (FACT-PCI), Perceived Cognitive Abilities (FACT-PCA), Impact of Perceived Cognitive Impairment on Quality of Life (FACT-QoL), and Comments from Others on Cognitive Function (FACT-others). Higher FACT-Cog scores indicate better cognitive functioning and quality of life.

We also evaluated education years, BMI, levels of anxiety, depression, and fatigue by Taiwanese versions of the Anxiety subscale of the Hospital Anxiety and Depression Scale (HADS-A) [24], the Patient Health Questionnaire (PHQ-9) [25], and the Brief Fatigue Inventory (BFI) [26]. Interleukins (IL) from helper T cell type 1 (Th1, including IFNγ, IL-12p70, IL-1β, IL-2, and TNFα), type 2 (Th2, including IL-4, IL-5, IL-10, and IL-13), and type 17 (Th17, including IL-5 and IL-17A) were measured because past literature has described how these cytokines may be regarded as biomarkers related to cancer, chemotherapy, neurotoxicity, mental illnesses [27], or contribute to cognitive deficits among cancer patients [7,28].

### 2.3. Statistical Analysis

Analysis of variance (ANOVA) was used to examine differences in patients’ characteristics and cognitive performance measures of three comparison groups: (i) pre-C/T, (ii) post-C/T, (iii) controls. Bonferroni correction was performed for post hoc tests. Since the distributions of cytokine levels were skewed to the right, these were transformed into log values for normality [10]. Analysis of covariance (ANCOVA) was performed to examine differences in log values of cytokines of the three subgroups while adjusting for significant covariates and cytokines.

Associations of cognitive performance and cytokine levels in the three subgroups while controlling for demographic (age, IQ, BMI, education years) and psychological (anxiety, depression, fatigue) covariates were modelled in stepwise multivariate linear regression analyses. A *p*-value of less than or equal to 0.05 was defined as a cut-off for further investigation since this study was planned to be exploratory.

When statistically significant correlations with neurocognitive function were found in the multivariate regression analysis, individual effects were examined in the post-hoc analysis after controlling for all other covariates. Further secondary multivariate regression analyses included comparing subgroups of breast cancer (pre-C/T and post-C/T subgroups) and controls as the ‘cancer vs. non-cancer’ comparison; post-C/T and control subgroups as ‘chemotherapy vs. non-cancer’ comparison; and pre-C/T and post C/T subgroups in the cancer group as ‘chemotherapy vs. non-chemotherapy’. Mediation analyses were performed to investigate whether mood symptoms or cytokines may mediate cognitive impairment differences between comparison groups.

## 3. Results

A total of 136 participants were included in this study: 106 BC patients, with 70 in the pre-C/T subgroup and 36 in the post-C/T subgroup, and 30 controls. Table 1 shows the demographic and clinical characteristics of our participants. The mean age of the combined sample was 50.5 (SD = 11.0) years. The pre-C/T subgroup was older, and had lower mean years of education and mean IQ scores than controls. The pre-C/T group also scored significantly higher on PHQ-9, HADS-A, and BFI-fatigue interference than controls or the post-C/T group. No significant differences were found in BMI between the three groups. Most of the BC patients were in Stage II. All patients in the post-C/T group had menopause (n = 36, 100%). Half of patients in the post-C/T subgroup received cyclophosphamide, 5-fluorouracil, epirubicin and doxorubicin, and docetaxel as the main chemotherapy regimen. More than 90% of post-C/T BC patients received a mastectomy. The range of intervals between surgery and recruitment was 93 to 288 days (Table 1).

As for levels of cytokines, IFN-γ, IL-12p70, and IL-17A were significantly lower in the pre-C/T and the post-C/T groups than in the controls (*p* = 0.001~0.004, Table 1). The plasma concentrations of IL-2 and IL-10 in the post-C/T group were lower than those in the control group (*p* = 0.016~0.036). IL-13 in the pre-C/T group was also lower than in the controls (*p* = 0.008). Table 2 showed that after controlling for subjects’ characteristics, mood status, and all other cytokines in ANCOVA, no significant differences in log-transformed values of cytokines were found between the three subgroups except for log- IFN-γ. Correlations between (serum) levels of cytokines and scores of cognitive domains are shown in supplementary Table S1 available online.

Results of subjective and objective neuropsychological tests are shown in Table 3. No significant differences were found in FACT-Cog test between the 3 subgroups. The pre-C/T and the post-C/T subgroups had significantly poorer performance than controls on verbal fluency (SFT; *p* = 0.002, *η*^2^ = 0.090).

After adjusting for mood and IQ using ANCOVA, significantly poorer performance in SFT persisted in the pre-C/T (*p* = 0.027) and post-C/T (*p* = 0.015) groups compared to controls (Table 4), and significantly better FACT-cog, perceived cognitive impairments or cognitive abilities were found in the pre-C/T group than in the controls. Levels of cognitive performances from other neuropsychological tests were similar between the three groups (Table 4).

Table 5 describes results from stepwise multivariate regression models of cytokines, patient characteristics, and mood status as independent variables and covariates, and FACT-Cog, FACT-PCI, FACT-PCA, or SFT as dependent variables for each of the three participant groups. Anxiety was associated with FACT-Cog in controls, and FACT-PCA in the control and post-C/T groups, with higher anxiety scores associated with higher perceived impairments. Similarly, higher depression scores were associated with more perceived impairments. Scores for fatigue were associated with FACT-Cog and FACT-PCI in the pre-C/T and control groups, and with FACT-PCA in the pre-C/T group. Higher age was associated with lower FACT-Cog and FACT-PCA in the post-C/T subgroup, and lower FACT-PCI in the pre- and post-C/T subgroups. The higher the BMI, the lower the FACT-PCI in the post-C/T subgroup. The higher the IQ, the higher the scores of FACT-PCA in the pre-C/T subgroup. The higher the Log IL5, the lower the FACT-PCI in the non-cancer and pre-C/T subgroups. The higher the Log IL10, the better the FACT-PCA in the non-cancer and pre-C/T subgroups. The higher the Log IL13, the lower the FACT-PCA in the pre-C/T subgroup.

For SFT, only education was associated with better performance of SFT in the control and pre-C/T groups. In the post C/T group, four factors were independent predictors of SFT: IQ and anxiety had significantly positive associations with SFT, while depression and log-IL-13 levels had significantly negative associations with SFT, i.e., the higher the log IL13, the poorer the performance on SFT. These four factors explained 7.7% of variance in SFT. Further examinations of effects of each cytokine also revealed that after controlling for all other covariates including significant cytokines, IL-13 was the only cytokine that was significantly negatively associated with SFT (β = −4.86, 95% CI: −8.83~−0.90, *p* = 0.018).

From the secondary multivariate regression model investigating significant factors associated with SFT among different comparisons, we found that when analyzing the subgroup of cancer vs. non-cancer (n = 136), positive associations were found for SFT and education years (β = 0.76, 95% CI: 0.43~1.10, *p* < 0.001) and anxiety (β = 0.9, 95% CI: 0.19~0.99, *p* = 0.005); negative associations were found for the group indicator of ‘cancer vs. non-cancer’ (β = −7.04, 95% CI: −10.34~−3.74, *p* < 0.001) and log IL-4 (β = −3.31, 95% CI: −6.51~−0.11, *p* = 0.043). As for the subgroup ‘chemotherapy vs. non-cancer’ (n = 66), SFT was found to be positively correlated with IQ (β= 0.26, 95% CI: 0.10~0.42, *p* = 0.002) and anxiety (β = 0.83, 95% CI: 0.10~1.57, *p* = 0.028); and negatively correlated with the group indicator of ‘chemotherapy vs. non-cancer’ (β = −5.92, 95% CI: −9.77~−2.06, *p* = 0.003). As for the subgroup ‘chemotherapy vs. non-chemotherapy’ (n = 106), SFT was found to be positively correlated with education years (β = 0.70, 95% CI: 0.34~1.07, *p* < 0.001) and anxiety (β = 0.15, 95% CI: 0.25~1.12, *p* = 0.002); and negatively correlated with log IL-4 (β = −4.76, 95% CI: −8.19~−1.32, *p* = 0.007).

**Figure 1:**
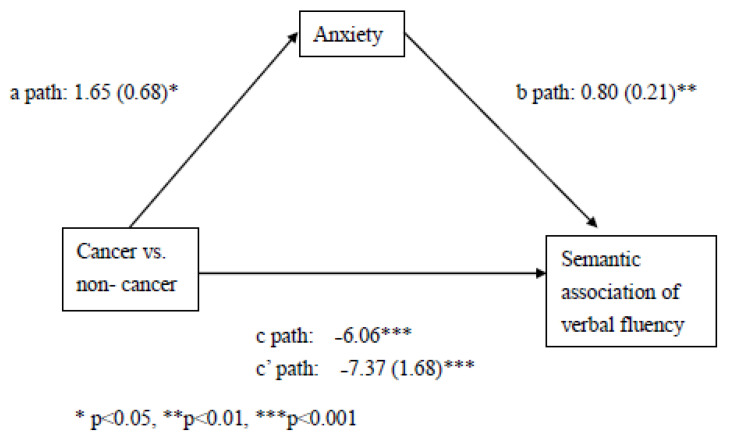
Results from mediation analysis for the associations of status of cancer vs. non-cancer (X), semantic association of verbal fluency (Y), partially mediated by anxiety (M).

Mediation analyses (Figure 1) showed that anxiety is a partial mediator of SFT and status of cancer vs. non-cancer (HADS scores coefficient = 0.80, t = 3.85, *p* < 0.001), but the status of chemotherapy vs. non-chemotherapy did not significantly predict SFT (coefficient = −1.58, t = −0.92, *p* = 0.36); and the status of chemotherapy vs. non-cancer did not predict anxiety (coefficient = 0.48, t = 0.74, *p* = 0.46).

## 4. Discussion

Our study is one of the first to investigate the association between cytokines and cognitive function controlling for IQ and psychological symptoms in patients with BC before and after chemotherapy, and with a non-cancer control group. We found most cognitive scores were not associated with cancer status or with cytokine levels. Despite this, a significantly poorer performance in verbal fluency (SFT) was found in the post-C/T group compared to non-cancer controls; and it may be explained by IQ, anxiety, and IL-13 from the multivariate regression model. The higher the log value of IL-13, the poorer the SFT was found in the post-C/T subgroup after further adjusting for significant cytokines. SFT was affected by the status of cancer more than chemotherapy. Significantly better self- perceived cognitive abilities were found in pre-C/T patients than in the controls, and this might be explained by fatigue, depression, anxiety, IL5, 10, or 13. While higher IL-10 was associated with better perceived cognitive ability in pre-C/T and control groups, higher IL-5 and IL-13 were associated with more severe subjective impairment in pre-C/T and control groups.

The result that post-C/T and pre-C/T cancer patients overall had poorer performance in the SFT than in the controls would be consistent with adverse effects of cancer and/or cytotoxic agents on cognitive function, as has been discussed in our previous paper [17] and other studies [4,15]. However, we feel that our findings suggest the poorer performance in SFT may be affected by cancer status than chemotherapy. This is because from our secondary analyses, while chemotherapy vs. non-cancer, and cancer vs. non-cancer were significantly associated with worse SFT, no significant association was found when comparing the chemotherapy vs. non-chemotherapy groups within the BC cases. Adding to the recent meta-analysis suggesting associations of verbal ability and caner status, visuospatial ability and chemotherapy [29], our finding also has some support from scant but relevant literature suggesting that memory impairments in newly diagnosed BC reflect cancer diagnosis rather than chemotherapy [3,10,30].

From our multivariate regression model in the post-C/T group, the finding that higher IL-13 predicted worse SFT may suggest impaired executive function after cancer or chemotherapy. Past studies regarding cognitive domain-specific predictors included associations of sTNFRI and short-term visual memory delayed match to sample test in BC patients receiving chemotherapy [13]. Cheung et al. reported correlations between plasma IL-1β and IL-4 with response speed [11]. Lyon et al. mentioned that there might be multiple relationships among cytokines and domain-specific cognitions, which varied over time [12]. The literature on our finding of IL-13 associations with learning and/or memory is still scarce and inconsistent [31,32], and no previous research has discussed its role in cancer-related cognitive impairment. In the peripheral system, IL-13 may promote allergic inflammation by T-helper type 2 (Th2); or be anti-inflammatory by down-regulating pro-inflammatory cytokines and T-helper type 1. In the central nervous system (CNS), they have been found to be potentially both neuroprotective or neurotoxic [32]. They may be protective and anti-inflammatory by promoting the M2 (or ‘healing’) microglia phenotype to repair neurons [32,33], or stimulate primary astrocytes to produce brain-derived neurotrophic factor associated with cognition [34]. IL-13 may also be pro-inflammatory, causing deaths of neurons sensitive to oxidation during neuro-inflammation [32]. Although no evidence to date has demonstrated IL-13 crossing the blood–brain barrier, rodent models have shown that IL-13 can be produced by microglia and neurons in the CNS, and may be enhanced by peripheral injections neurotoxins [32]. Longitudinal research on roles and interactions of ‘healing’ or ‘harmful’ effects of IL-13 on cognition in BC and chemotherapy may still be needed.

Considering subjective complaints, we found that pre-C/T patients had significantly higher scores in self-perceived cognitions than the controls, and lower IL-5 and IL-13 levels were associated with better perceived function in the FACT-PCI or FACT-PCA, respectively, in pre-C/T patients and controls. On the contrary, higher IL-10 levels were associated with worse subjective function in the FACT-PCA in pre-C/T patients and controls. Although the level of sTNFRII was found to be associated with subjective cognitive complaints in BC patients with or without chemotherapy [10], no previous reports were found in the associations of IL-5 or IL13 and subjective cognitive impairments. Recent research has mentioned IL-10 as an anti-inflammatory cytokine related to Alzheimer disease (AD) [35]. IL-10 is known for reducing immune and inflammatory responses and inhibits the expression of cytokine receptors [36,37]. Although the formation of senile plaque in AD might be associated with activated microglial cells and IL-10 gene polymorphism [38], it remains to be clarified whether anti-inflammatory responses in the brain from IL-10 contribute to neurodegeneration [35,39].

We did not find associations between other cytokines and cognitive declines in the 3 subgroups. Although chemotherapy is generally considered to be immunosuppressive, responses from plasma concentrations, types, or patterns of cytokines may change with time, or have different reactions or interactions [12,40,41]. For instance, it is interesting that all cytokines in the control group were higher than those in the pre-C/T and post-C/T groups. It is possible that the lower cytokine levels in the pre-C/T group compared to controls may be due to immune escape; as Bates et al. described in their review that the development of breast cancer may be related to mechanisms that decrease the ability of immune recognitions to cancer cells, as well as reducing the promotions of immunosuppression. In post-C/T patients, the lower cytokine levels compared to controls may be due to effects from chemotherapy treatments. Lyon et al. described the increase of IL-6 during chemotherapy but decrease afterwards; while continuous decline from baseline over time were for IL-17 [12]. Alterations in different cytokines during inflammatory responses induced by cancer or chemotherapy might be responsible for cognitive deficits in our post-C/T patients [30,42,43]. However, it is important to remember that our samples were relatively small and cytokine levels may be too subject to external influence resulting in loss of signal.

Finally, the status of menopause was not included in the multivariate regression analysis because univariate analysis of menopause and verbal fluency in the post-C/T group was not able to perform when everyone had menopause. Nearly half of these patients had menopause naturally (n = 15), while 15 of them had menopause after chemotherapy, 4 had menopause after hysterectomy, 1 after oophorectomy, and 1 after hormone therapy. No significant correlations were found for reasons of menopause and verbal fluency (*p* = 0.990). In the subsample that included only non-cancer controls and BC patients without chemotherapy, the status of menopause was not associated with verbal fluency (*p* = 0.079). Therefore, we believe that impairments in verbal fluency were more likely to be associated with chemotherapy, not the status or reasons of menopause.

### Strengths and Limitations

Major strengths of this study are the comprehensive neuropsychological assessments using well-validated batteries and structured ascertainment of IQ, self-perceived, and objective cognitive impairment. The key limitation is the study’s cross-sectional design that restricted the exploration of possible causal processes. Further long-term cohort study investigating changes in cognitive functions before and after chemotherapy are still warranted to control more intrinsic and extrinsic factors to establish relevant causal mechanisms. Second, our numbers of healthy control and post-C/T BC patients may be insufficient to detect small associations between cytokine levels and cognitive performance. Third, this study was conducted in Taiwanese patients and the generalizability to other populations may be restricted. Fourth, our study was still restricted by a limited set of biomarkers, as well as lacking information on some potential confounders, such as diet or physical activity.

## 5. Conclusions

Our main findings suggest that performance in verbal fluency, at least, might be affected by the presence of BC and mediated by anxiety. Higher levels of cytokines IL-5 and IL-13 were significantly associated with lower subjective cognitive complaints and lower verbal fluency after controlling for IQ and psychological factors. Higher IL-10, on the other hand, was associated with better subjective perceived cognition. Our results indicated that semantic association of verbal fluency and anxiety may be used as important information for providing additional related psychosocial managements in BC patients after chemotherapy. Relevant prophylactic interventions for cognitive preservation associated with regulations in cytokines might also need to be further explored.

## Figures and Tables

**Table 1:** Summary of subject characteristics.

Characteristics, Cognitive Status, and Cytokine Levels	Pre-C/T	Post-C/T	Non-Cancer	ANOVA		
	Cancer Patients	Cancer Patients	Controls			
	(*N* = 70)	(*N* = 36)	(*N* = 30)			
Basic Information	Mean (SD)	Mean (SD)	Mean (SD)	*p*	*η* ^2^	Post Hoc Tests ^‡^
Age (years)	51.74 (11.39)	49.97 (10.04)	48.13 (11.30)	0.312	0.017	
BMI	24.14 (3.73)	24.42 (4.15)	24.77 (3.00)	0.738	0.005	
Education (years)	11.01 (4.46)	11.97 (3.82)	12.50 (3.97)	0.220	0.023	
IQ ^†^	100.00 (11.37)	101.14 (13.66)	106.60 (10.43)	0.039 *	0.049	control > pre-C/T
PHQ-9	5.14 (4.42)	4.06 (3.91)	2.07 (2.56)	0.002 *	0.088	pre-C/T > control
HADS-A	4.21 (3.71)	2.44 (2.56)	1.97 (2.68)	0.002 *	0.090	pre-C/T > control
BFI—fatigue severity score	1.43 (2.29)	2.26 (2.14)	2.29 (2.62)	0.108	0.033	
BFI—fatigue interference score	4.04 (6.23)	4.94 (8.04)	0.68 (1.36)	0.014 *	0.063	pre-C/T > control post-C/T > control
	*N* (*%*)	*N* (*%*)	*N* (*%*)			
Menopausal status (No)	37 (52.9)	36 (100)	14 (46.7)	<0.001		
Stage				0.823		
Stage I	21 (30.00)	12 (30.00)	-			
Stage II	36 (51.43)	16 (44.44)	-			
Stage III	12 (17.14)	7 (19.44)	-			
Treatment						
Mastectomy	17 (24.64)	33 (94.29)	-			
Time from Mastectomy to recruitment, days, Mean (SD)	93.53 (181.55)	288.43 (56.84)				
Radiotherapy	1 (1.43)	30 (83.33)	-			
Hormonal therapy	3 (4.29)	28 (77.78)	-			
Targeted therapy	0	0	-			
Chemotherapy regimen						
Cyclophosphamide, 5-fluorouracil, epirubicin and doxorubicin, and docetaxel	-	15 (50.00)	-			
Cyclophosphamide, 5-fluorouracil, epirubicin and doxorubicin, docetaxel, and cisplatin	-	4 (13.33)	-			
Cyclophosphamide, 5-fluorouracil, epirubicin and doxorubicin	-	4 (13.33)	-			
Cyclophosphamide, methotrexate, and 5-fluorouracil	-	3 (1.00)	-			
Cyclophosphamide, methotrexate, epirubicin and doxorubicin	-	1 (0.03)	-			
Functional Assessment of Cancer Therapy Cognitive Scale (FACT-Cog)	119.26 (10.54)	118.58 (10.11)	116.13 (16.25)	0.485	0.011	
Perceived Cognitive Impairment	66.03 (6.41)	65.83 (6.25)	64.50 (9.70)	0.615	0.007	
Comments from Others	15.71 (0.89)	15.56 (0.81)	15.27 (1.82)	0.201	0.024	
Perceived Cognitive Abilities	22.13 (3.73)	21.81 (3.48)	21.30 (4.79)	0.624	0.007	
Impact of Perceived Cognitive Impairments on Quality of Life	15.39 (1.96)	15.39 (1.42)	15.07 (2.16)	0.714	0.005	
Cytokines						
Th1						
IFNγ	6.35 (5.68)	6.72 (4.62)	12.45 (7.32)	<0.001 *	0.156	control > pre-C/T control > post-C/T
IL-12p70	1.90 (1.56)	1.93 (1.42)	3.25 (2.00)	0.001	0.016	control > pre-C/T control > post-C/T
IL-1β	0.74 (0.81)	0.64 (0.49)	0.95 (0.50)	0.175	0.026	
IL-2	1.29 (1.10)	0.96 (0.80)	1.66 (1.29)	0.036 *	0.049	control > post-C/T
TNF	4.38 (2.55)	4.99 (2.58)	5.61 (2.83)	0.093	0.035	
Th2						
IL-4	10.09 (11.11)	14.75 (22.05)	18.62 (18.01)	0.047	0.045	
IL-5	1.53 (1.22)	1.16 (0.95)	1.70 (1.18)	0.134	0.030	
IL-10	4.64 (3.83)	3.45 (3.34)	6.28 (4.83)	0.016 *	0.060	control > post-C/T
IL-13	2.56 (2.71)	2.84 (4.03)	4.68 (2.72)	0.008 *	0.071	control > pre-C/T
Th17						
IL-6	1.43 (1.68)	1.63 (1.88)	2.33 (1.69)	0.060	0.041	
IL-17A	5.40 (3.97)	4.87 (2.65)	7.70 (3.93)	0.004 *	0.078	control > pre-C/T control > post-C/T

^†^ Intelligence Quotient (IQ) estimated by the short form of the Taiwan Wechsler Adult Intelligence Scale-III; PHQ-9: Patient Health Questionnaire-9; HADS-A: Hospital Anxiety Depression Scale; BFI: Brief Fatigue Inventory; ^‡^ Significant difference after Bonferroni correction; * *p* < 0.05.

**Table 2:** Analysis of covariance (ANCOVA) in patients’ characteristics and log-transformed values of cytokines among the three groups.

Characteristics and Log Transformed Values of Cytokines	Pre-C/T	Post-C/T	Non-Cancer	ANCOVA		
	Cancer Patients	Cancer Patients	Controls			
	(*N* = 68)	(*N* = 35)	(*N* = 30)			
	Mean (SD)	Mean (SD)	Mean (SD)	*F*	*η* ^2^	*p*
IQ ^†^	100.00 (11.37)	101.14 (13.66)	106.60 (10.43)	2.907	0.048	0.059
PHQ-9	5.01 (4.40)	3.66 (3.13)	2.07 (2.56)	5.399	0.085	0.054
HADS-A	4.21 (3.74)	2.37 (2.56)	1.97 (2.68)	4.84	0.077	0.010 * Pre-C/T > control
BFI—fatigue severity score	1.38 (2.24)	2.18 (2.12)	2.29 (2.62)	8.165	0.123	<0.001 Pre-C/T < control Pre-C/T < post-C/T
BFI—fatigue interference score	4.04 (6.23)	4.94 (8.04)	0.68 (1.36)	3.352	0.055	0.038 Post-C/T >control
Cytokines						
Log_IFNr	0.59 (0.47)	0.67 (0.43)	1.01 (0.31)	3.052	0.048	0.046 * pre C/T < non-cancer
Log_IL-2	−0.01 (3.42)	−0.20 (0.43)	0.09 (0.40)	2.612	0.041	0.078
Log_TNFα	0.57 (0.25)	0.65 (0.19)	0.71 (0.18)	1.012	0.017	0.367
Log_IL-4	0.78 (0.43)	0.93 (0.45)	1.12 (0.37)	3.193	0.051	0.045
Log_IL-12	0.13 (0.39)	0.11 (0.45)	0.41 (0.34)	0.543	0.009	0.582
Log_IL-10	0.51 (0.40)	0.31 (0.50)	0.61 (0.48)	2.609	0.042	0.078
Log_IL-13	0.15 (0.54)	0.10 (0.67)	0.57 (0.35)	2.123	0.034	0.124

Adjusted for IQ, PHQ-9, HADS-A, fatigue, and significant cytokines; ^†^ Significant difference after Bonferroni correction; * *p* < 0.05.

**Table 3:** Mean scores on subjective and objective cognitive evaluations (ANOVA).

Subjective and Objective Cognitive Assessments	Pre-C/T Cancer Patients (*N* = 68)	Post-C/T Cancer Patients (*N* = 35)	Non-Cancer Controls (*N* =30)	ANOVA		
	Mean (SE)	Mean (SE)	Mean (SE)	*p*	*η^2^*	Post Hoc Tests ^‡^
Functional Assessment of Cancer Therapy Cognitive Scale (FACT-Cog)	119.26 (10.54)	118.58 (10.11)	116.13 (16.25)	0.485	0.011	
Perceived Cognitive Impairment	66.03 (6.41)	65.83 (6.25)	64.50 (9.70)	0.615	0.007	
Comments from Others	15.71 (0.89)	15.56 (0.81)	15.27 (1.82)	0.201	0.024	
Perceived Cognitive Abilities	22.13 (3.73)	21.81 (3.48)	21.30 (4.79)	0.624	0.007	
Impact of Perceived Cognitive Impairments on Quality of Life	15.39 (1.96)	15.39 (1.42)	15.07 (2.16)	0.714	0.005	
Attention function						
Digit Span	11.25 (2.97)	11.02 (3.38)	12.01 (2.92)	0.393	0.014	
Color Trails Test 1	51.52 (19.99)	49.26 (22.89)	47.27 (19.05)	0.623	0.007	
Executive function						
Semantic Association of Verbal Fluency	38.91 (8.22)	37.33 (8.65)	44.43 (8.22)	0.002 *	0.090	Control > pre-C/T Control > post-C/T
Orthographical Fluency Test	17.59 (7.71)	17.60 (7.82)	20.44 (5.84)	0.178	0.026	
Color Trails Test 2	98.20 (34.75)	98.84 (35.08)	88.67 (29.77)	0.380	0.014	
Memory function						
Word List—Total immediate recall	9.94 (2.50)	9.67 (2.77)	10.93 (2.94)	0.132	0.030	
Word List—Long-delay recall	10.59 (2.55)	10.61 (2.53)	10.97 (2.68)	0.782	0.004	
Word List—Recognition	11.07 (2.39)	11.44 (2.27)	11.23 (2.21)	0.734	0.005	
Visuospatial construction						
Block Design	9.33 (3.00)	8.75 (3.38)	9.67 (3.20)	0.480	0.011	
Processing speed						
Digit Symbol Substitution	10.26 (2.73)	10.36 (2.66)	11.13 (2.93)	0.335	0.016	
Prospective memory						
Event-based	4.07 (1.32)	3.87 (0.32)	3.87 (0.43)	0.487	0.011	
Time-based	3.34 (0.84)	3.34 (0.79)	3.53 (0.73)	0.517	0.010	

^‡^ Significant difference after Bonferroni correction; * *p* < 0.05.

**Table 4:** Mean scores of subjective and objective cognitive evaluations after adjusting for depression, anxiety, and fatigue (ANCOVA).

Subjective and Objective Cognitive Assessments	Pre-C/T Cancer Patients (*N* = 68)	Post-C/T Cancer Patients (*N* = 35)	Non-Cancer Controls (*N* =30)	ANCOVA ^†^		
	Mean (SD)	Mean (SE)	Mean (SE)	*p*	*η* ^2^	Post Hoc Tests ^‡^
Functional Assessment of Cancer Therapy Cognitive Scale (FACT-Cog)	119.29 (10.67)	119.26 (9.40)	116.13 (16.25)	0.006	0.077	Pre-C/T > Control
Perceived Cognitive Impairment	66.00 (6.48)	66.29 (5.71)	64.50 (9.70)	0.031	0.054	Pre-C/T > Control
Comments from Others	15.71 (0.90)	15.60 (0.78)	15.27 (1.82)	0.052	0.046	
Perceived Cognitive Abilities	22.21 (3.74)	21.94 (3.43)	21.30 (4.79)	0.019	0.061	Pre-C/T > Control
Impact of Perceived Cognitive Impairments on Quality of Life	15.38 (1.99)	15.43 (1.42)	15.07 (2.16)	0.058	0.044	
Attention function						
Digit Span	11.31 (2.85)	11.06 (3.42)	12.01 (2.92)	0.440	0.013	
Color Trails Test 1	51.36 (19.93)	48.80 (23.06)	47.27 (19.05)	0.623	0.007	
Executive function						
Semantic Association of Verbal Fluency	38.99 (8.29)	37.43 (8.76)	44.43 (8.22)	0.011 *	0.070	Control > pre-C/T Control > post-C/T
Orthographical Fluency Test	17.79 (7.70)	17.91 (7.72)	20.44 (5.84)	0.708	0.005	
Color Trails Test 2	97.16 (34.24)	97.80 (35.02)	88.67 (29.77)	0.697	0.006	
Memory function						
Word List—Total immediate recall	9.96 (2.52)	9.69 (2.81)	10.93 (2.94)	0.693	0.006	
Word List—Long-delay recall	10.57 (2.58)	10.69 (2.53)	10.97 (2.68)	0.709	0.005	
Word List—Recognition	11.09 (2.42)	11.43 (2.31)	11.23 (2.21)	0.504	0.011	
Visuospatial construction						
Block Design	9.43 (2.94)	8.74 (3.43)	9.67 (3.20)	0.086	0.038	
Processing speed						
Digit Symbol Substitution	10.26 (2.72)	10.40 (2.69)	11.13 (2.93)	0.975	0.000	
Prospective memory						
Event-based	4.13 (1.24)	3.89 (0.33)	3.87 (0.43)	0.115	0.034	
Time-based	3.34 (0.84)	3.34 (0.80)	3.53 (0.73)	0.328	0.018	

Adjusted for IQ, anxiety, depression, and fatigue. ^‡^ Significant difference after Bonferroni correction; * *p* < 0.05.

**Table 5:** Summary of multivariable regression analyses in FACT- Cog total scores, FACT- PCI, FACT- PCA, SFT among the three groups.

Predictors	Non cancer (*n* = 30)						Pre-C/T (*n* = 68)						Post-C/T (*n* = 35)			
	β	R^2^	ΔR^2^	*p*-Value	Confidence Interval (CI)		β	R^2^	ΔR^2^	*p*-Value	CI	β	R^2^	ΔR^2^	*p*-Value	CI
**FACT-Cog**		0.599	0.099	0.012				0.155	0168	0.001	0.33~1.19		0.429	0.212	0.001	
Age												−0.43			0.001	−0.68~−0.18
HADS	−2.08			0.012	−3.68~	−0.49										
PHQ-9												−1.48			0.001	−2.27~−0.68
BFI_interference	−7.01			<0.001	−10.16~	−3.85	−0.73				−1.13~−0.33					
**FACT-PCI**		0.569	0.084	0.025				0.334	0.058	0.019			0.397	0.078	0.044	
Age							0.16			0.011	0.04~0.27	−0.22			0.007	−0.37~−0.06
BMI												−0.40			0.044	−0.78~−0.01
PHQ-9							−0.40			0.019	−0.72~−0.07	−0.71			0.008	−1.23~−0.20
BFI_interference	−4.83			<0.001	−6.63~	−3.03	−0.36			0.005	−0.61~−0.11					
Log IL-5	−7.98			0.025	−14.87~	−1.08	−0.73				−1.13~−0.33					
**FACT-PCA**		0.443	0.115	0.021				0.227	0.069	0.017			0.340	0.144	0.010	
Age												−0.17			0.001	−0.27~−0.08
IQ							0.01			0.005	0.03~0.17					
PHQ							−0.23			0.017	−0.41~−0.04					
HADS	−0.76			0.010	−1.32~	−0.02						−0.51			0.010	−0.89~−0.13
BFI_interference	−1.32			0.021	−2.43~	−0.21										
Log IL-10	3.88			0.011	0.97~	6.79	3.66			0.002	1.43~5.89					
Log IL-13							−2.28			0.008	−3.95~−0.62					
**SFT**		0.218	0.245	0.005				0.144	0157	0.001			0.458	0.077	0.036	
Years of education	1.02				0.32~	1.72	0.76				0.33~1.19					
IQ												0.27			0.002	0.11~0.44
HADS-A												1.87			0.001	0.86~2.87
PHQ-9												−0.90			0.036	−1.73~−0.06
Log IL-13												−4.94			0.008	−8.46~−1.42

Multivariate stepwise regression adding all variables (including cytokines) into the model. FACT-Cog: Functional Assessment of Cancer Therapy Cognitive Function; FACT-PCI: Perceived Cognitive Impairment; FACT-PCA: Perceived Cognitive Abilities; β: standardized regression coefficients; R^2^ = R square; ΔR^2^ = R square change.

## Data Availability

The data presented in this study are available on request from the corresponding author. The data are not publicly available due to privacy and ethical issues.

## Supplementary Materials

The following are available online at https://www.mdpi.com/article/10.3390/cancers13112576/s1, Table S1: Correlations between levels of cytokines and scores of cognitive domains in post-C/T group.
