# Supplementary material for: Effects of Cancer, Chemotherapy, and Cytokines on Subjective and Objective Cognitive Functioning Among Patients with Breast Cancer
Source: Cancers (Basel). 2021 May 24;13(11):2576. doi: 10.3390/cancers13112576 (PMC8197334; doi:10.3390/cancers13112576)
Supplement: Supplementary file 1 [file cancers-13-02576-s001.zip › cancers-1164134-supplementary.pdf]

Supplementary Table s1. Correlations between levels of cytokines and scores of cognitive domains in post-C/T group.

Neurocognitive tests	Serum levels of cytokines in the Post- C/T group (n=35)										
	IFNr	IL_12p70	IL_1B	IL_2	TNFa	IL_4	IL_5	IL_10	IL_13	IL_6	IL_17A
**Attention function**											
Digit Span	-0.19	-0.34	-0.41*	-0.35	-0.14	-0.45*	-0.19	-0.28	-0.15	-0.24	-0.14
Color Trails Test 1	-0.29	-0.22	-0.24	-0.27	0.05	-0.25	-0.41*	-0.17	-0.15	-0.01	-0.41*
**Executive function**											
Semantic	0.09	-0.06	-0.15	-0.23	-0.19	-0.38*	-0.25	-0.1	-0.44*	-0.37*	0.08
Association of Verbal Fluency	0.63	0.76	0.42	0.22	0.31	0.04	0.18	0.6	0.01	0.04	0.68
Orthographical Fluency Test	-0.07	0.19	0.03	-0.25	0.24	-0.06	-0.01	-0.02	0.14	0.07	-0.12
Color Trails Test 2	-0.32	-0.25	-0.17	-0.27	0.1	-0.28	-0.36*	-0.03	-0.38*	0.25	-0.38*
**Memory function**											
Word List - Total immediate recall	-0.21	-0.2	-0.35	-0.36*	-0.22	-0.36*	-0.27	-0.13	-0.33	-0.09	-0.11
Word List - Long-delay recall	0.02	-0.01	-0.20	-0.08	-0.20	-0.16	0.10	0.08	-0.23	-0.13	0.22
Word List - Recognition	0.04	-0.01	0.04	-0.13	0.07	-0.01	0.11	-0.08	-0.08	0.13	0.06
**Visuospatial construction**											
Block Design	0.27	-0.06	0.01	0.14	-0.05	-0.12	0.00	0.09	-0.24	-0.27	0.40*
**Processing speed**											
Digit Symbol Substitution	-0.01	-0.12	-0.19	-0.22	-0.30	-0.23	-0.13	-0.14	-0.32	-0.28	0.12
**Prospective memory**											
Event-based	0.22	0.35	0.25	0.06	0.25	0.27	0.15	0.20	0.14	0.21	0.10
Time-based	0.14	-0.05	-0.13	0.16	-0.32	-0.14	0.29	0.35	-0.12	-0.11	0.25
**Functional Assessment of Cancer Therapy Cognitive Scale (FACT- Cog)**											
Sum	-0.01	0.10	0.15	0.02	0.07	0.14	0.08	0.01	0.25	0.11	0.00
Perceived Cognitive Impairment	-0.09	-0.07	0.02	-0.05	0.04	0.11	0.16	-0.10	0.22	0.01	-0.04
Comments from Others	-0.26	-0.29	-0.21	-0.26	-0.15	-0.13	-0.03	-0.07	0.07	0.06	-0.16
Perceived Cognitive Abilities	0.10	0.29	0.27	0.10	0.08	0.20	-0.03	0.10	0.24	0.17	0.05
Impact of Perceived Cognitive Impairments on Quality of Life	0.06	0.12	0.13	0.10	0.17	-0.15	0.04	0.22	0.03	0.09	0.07

* $p < 0.05$.
